# Supplementary material for: A portable feedback-controlled pump for monitoring eye outflow facility in conscious rats
Source: PLoS One. 2023 Jan 11;18(1):e0280332. doi: 10.1371/journal.pone.0280332 (PMC9833506; doi:10.1371/journal.pone.0280332)
Supplement: S1 Appendix — (DOCX) [file pone.0280332.s001.docx]

*Supporting Information for “A portable feedback-controlled pump for monitoring eye outflow facility in conscious rats”*

**Pump operation as a constant pressure source**

A key aspect of system design is the operation and programmatic control of a pump. A design challenge in delivering fluid at microfluidic flow rates of known value is that pump output depends on two factors. One is the pump speed, which is expressed here in terms of the percentage of time that the pump is on during a power cycle (i.e., duty cycle). The other is pump load, which is the collective impedance of all elements through which pump output flows such as the eye, cannula, and connective tubing. To better understand this multifactorial dependence, Fig S1A depicts pump pressure head P_P_ and flow F_P_ according to manufacturer specifications for duty cycles ranging from 25% to 100% and for a load of arbitrary impedance. Note that F_P_ decreases linearly with increasing P_P_ for a given duty cycle, while it increases linearly with P_P_ for the arbitrary load impedance. The intersect of the two relations specifies the system operating point for a given duty cycle and output impedance. This means that running the pump at 25, 50, 75, and 100% duty cycle against an arbitrary load of 0.1 mmHg⋅min⋅μl^-1^ would produce P_P_ of 27, 55, 83, and 110 mmHg and F_P_ of 0.28, 0.56, 0.84, and 1.12 μl⋅min^-1^, respectively, per the manufacturer. A much larger load would be needed to generate microinfusion rates on par with aqueous humor production, so Fig S1B plots P_P_ at 100% duty cycle for a wide range of load impedances R_S_ based on manufacturer specs. Note that P_P_ saturates around 200 mmHg for R_S_ greater than 5 mmHg⋅min⋅μl^-1^, indicating that the pump should act like a constant pressure source in this regime. To validate the idea, the pump was loaded by different flow restrictors that were fabricated by hand to have impedances between 20 to 200 mmHg⋅min⋅μl^-1^. Fig S1C plots measured P_P_ versus pump duty cycle for several such restrictors. As predicted, P_P_ depended only on duty cycle and was insensitive to differences in R_S_. A high-impedance flow restrictor was thereby added to the system to capitalize on this property and minimize the impact on pump output of eye impedance and downstream system impedances that can vary between animals or across experiments.


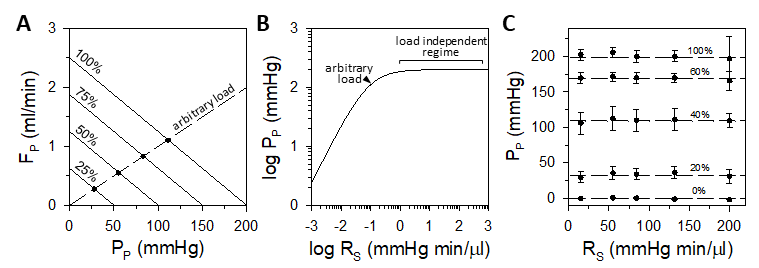


**Fig S1.** **Pump operation as a constant pressure source.** (A) Flow versus pressure curves provided by the pump manufacturer for multiple duty cycles (solid lines) and for an arbitrary load (dashed line). The load resistance of 0.1 mmHg⋅min⋅μl^-1^ is used for illustrative purpose only. Dots indicate pump output at each duty cycle for such a load. (B) Pump pressure head versus load resistance based on manufacturer specs for a 100% duty cycle. The dot corresponds to the arbitrary load of 0.1 mmHg⋅min⋅μl^-1^. (C) Empirical measurements of pump pressure head when loaded by flow restrictors of widely different impedance. Dashed lines indicate the behavior of a constant pressure source. Error bars give standard deviation.
