# Supplementary material for: A portable feedback-controlled pump for monitoring eye outflow facility in conscious rats
Source: PLoS One. 2023 Jan 11;18(1):e0280332. doi: 10.1371/journal.pone.0280332 (PMC9833506; doi:10.1371/journal.pone.0280332)
Supplement: Attachment — Submitted filename: response2.docx [file pone.0280332.s004.docx]

**Response to Comments (PONE-D-22-19808)**

We thank the reviewers for examining the manuscript a second time. Reviewer #2 has no further comments. Below is our response to the few remaining concerns raised by Reviewer #1.

Reviewer #1

1. R_G element in Figure 2A. We asked that this be removed but the authors wanted to retain it, stating that it must be present because "the globe does not instantaneously gain or lose volume following step changes in pressure". While it is true that the globe does not instantaneously change in volume, the inference that R_G is required to explain this behavior is categorically false. More specifically, the globe compliance interacting with the outflow resistance and/or needle resistance creates a RC time constant that can explain globe filling dynamics. The only reason I can imagine to include R_G is to represent the viscoelastic response of the corneoscleral shell, in which case I guess the shell is being represented as a Maxwell body. I doubt the system has the sensitivity to measure this. Rather than having this extremely confusing element in the diagram which is never used, why not just delete it???

*Response: We have removed the R_G element from the model in Figure 2A.*

2. Suggest moving Fig. 2B-C to supplemental. Characterization of the pump with arbitrary load is not essential for readers to understand the paper and is confusing given that the load shown is not relevant to aqueous humor drainage in rats. It may be useful for a small subset of people interested in the specific pump dynamics, but appearing in the supplemental material is fine for that purpose.

*Response: We have moved Fig 2B-2D and the accompanying text on pump operation and how it was made into a constant pressure source to a new Supplemental Information section (S1 Appendix).*
